# 

**Published:** 1910-10-15

**Authors:** 


### CONTENTS.

| | PAGE |
|---|---|
| **The Nationalisation of the Profession** ... ... ... | 63 |
| **The Ethnology of the American Medical Profession** | 64 |
| **Annotations—** | |
| The late Professor von Leyden—The Portuguese Revolution—The Cholera Scare ... ... ... ... ... ... | 65 |
| **Medical Opinion and Movement** ... ... ... ... | 66 |
| **Hospital Clinics—** | |
| Inflammation of the Iris. By A. Maitland Ramsay, M.D., F.R.F.P.S.G. ... ... ... ... ... ... ... | 69 |
| **Special Articles—** | |
| The Modern Treatment of Fractures ... ... ... ... ... | 71 |
| An Epitome of Pediatrics... ... ... ... ... ... ... | 73 |
| **Practical Notes on Diagnosis and Treatment** ... | 74 |
| **"The Hospital" Medical Book Supplement—No. XXXV.** ... ... ... ... ... ... ... | 75 |
| **News and Events of the Week** ... ... ... ... | 79 |
| **Appointments and Resignations** ... ... ... ... | 80 |
| **Obituary** ... ... ... ... ... ... ... ... | 80 |
| **The Week's Work—** | |
| Royal Hospitals—The Jewish Hospital Scheme—The Fire at Leeds Infirmary—This Week's Drug Market ... ... ... | 81 |
| **Parliament and Publications—** | |
| The Cost of Poor-Law Medical Relief—The Sale of Proprietary Medicines ... ... ... ... ... ... ... ... ... | 82 |
| **Special Institutional Articles—** | |
| The Social Policy of Voluntary Hospitals ... ... ... ... | 83 |
| **Hospital Contracts and Prices** ... ... ... ... ... | 89 |
| **The Hospital World** ... ... ... ... ... ... ... | 90 |
| **Gifts and Bequests** ... ... ... ... ... ... ... | 90 |
| **Jottings** ... ... ... ... ... ... ... ... ... | 90 |
| **Institutional Notes and News** ... ... ... ... ... | 91 |
| **New Appliances and Things Medical** ... ... ... | 92 |
| **Editor's Letter-Box—** | |
| The Heritage School of Arts and Crafts for Crippled Boys, Chailey, Sussex ... ... ... ... ... ... ... ... | 92 |

